# Analysis of Nonstationary Radiometer Gain Using Ensemble Detection

**DOI:** 10.1109/jstars.2020.2993765

**Published:** 2020

**Authors:** Mustafa Aksoy, Hamid Rajabi, Paul E. Racette, John Bradburn

**Affiliations:** University at Albany, State University of New York, Albany, NY 12222 USA; University of California, Merced, CA 95343 USA; NASA Goddard Space Flight Center, Greenbelt, MD 20771 USA; University at Albany, State University of New York, Albany, NY 12222 USA

**Keywords:** Autoregressive AR(1) model, ensemble detection, nonstationary radiometer gain, radiometer calibration

## Abstract

Radiometer gain is generally a nonstationary random process, even though it is assumed to be strictly or weakly stationary. Since the radiometer gain signal cannot be observed independently, analysis of its nonstationary properties would be challenging. However, using the time series of postgain voltages to form an ensemble set, the radiometer gain may be characterized via radiometer calibration. In this article, the ensemble detection algorithm is presented by which the unknown radiometer gain can be analytically characterized when it is following a Gaussian model (as a strictly stationary process) or a 1st order autoregressive, AR(1) model (as a weakly stationary process). In addition, in a particular radiometer calibration scheme, the nonstationary gain can also be represented as either Gaussian or AR(1) process, and parameters of such an equivalent gain model can be retrieved. However, unlike stationary or weakly stationary gain, retrieved parameters of the Gaussian and AR(1) processes, which describe the nonstationary gain, highly depend on the calibration setup and timings.

## I. Introduction

RADIOMETERS are widely used to measure geophysical parameters to examine variations in the earth and planetary systems. These measurements typically need to be obtained over large temporal or spatial scales. However, enhanced radiometric accuracy and sensitivity are required in microwave radiometry, as an accurate radiometer facilitates the possibility of high-resolution contrasts in variations of physical parameters from the rest of the measured noise. For instance, the retrieval of geophysical parameters, such as precipitable water vapor, ocean surface salinity, wind measurements, and liquid and ice water paths require enhanced accuracy and finer resolution [1]–[5]. Thus, given the increasing importance of radiometer calibration in deriving greater geophysical information from radiometer measurements, a precise calibration process should be obtained to detect long-term variations in such variables [6]–[13].

Radiometers are calibrated on board using a set of calibration targets with known temperatures. Although one can better overcome the error due to fluctuations or nonlinearities by using three or more targets [14]–[18], calibration is usually performed by measuring one cold and one hot target to calculate the radiometer gain and offset [19]–[22]. Then, the radiometer gain and offset are used to convert the measured voltage signals (as emitted power from an unknown source) into corresponding estimated antenna temperatures. In this article, radiometer systems and their calibration are examined based upon the uncertainty in estimated antenna temperature according to what Racette and Lang discussed in [23] and [24]. This uncertainty is highly dependent on calibration parameters, i.e., the observation time of calibration targets, receiver temperature, bandwidth, integration time, number of calibration targets, and associated calibration target temperatures.

The radiometer gain is not independently observed and can only be extracted as a result of the radiometer calibration process. Furthermore, since it is usually a realization of a nonstationary random process, it may reveal different characteristics under different calibration setups. On the other hand, the time series of postgain voltages associated with the calibration targets form an ensemble set for the underlying gain process from which the nonstationary radiometer gain can be characterized via ensemble detection [25]. Ensemble detection is a new technique that utilizes ensemble sets to characterize and analyze nonstationary radiometer gain based on the calibration structure and evaluates calibration errors and uncertainties due to temporal variations in the radiometer response. The primary objective of this article is to introduce and validate this technique by characterizing several radiometer gain processes both analytically and numerically for various calibration setups with known uncertainty levels for calibrated antenna temperature estimates conditioned such that the gain is following a strictly or weakly stationary random process. The same methodology is then implemented to examine radiometer gain described by nonstationary processes. Specifically, the main components of this article are summarized as follows.

1. First, ensemble detection is introduced and applied to a given radiometer calibration scheme assuming two different types of radiometer gain in terms of their stationarity: a strictly stationary Gaussian random process and a weakly stationary 1st order autoregressive, AR(1), random process.
2. Then, analytical models for the relationship between the parameters of the estimated antenna temperature and the radiometer gain process are mathematically derived for each of these two models.
3. These models are utilized in order to characterize unknown Gaussian and AR(1) gain radiometer processes in several calibration setups.
4. The same procedure is used to characterize a nonstationary gain process. In this case, it is shown that, for a given calibration structure, an equivalent stationary or weakly stationary gain model can be found, and the characteristics of the equivalent gain process vary depending on the calibration timing and setup.

The rest of the article is organized as follows. In Section II, ensemble detection is described. Sections III and IV discuss how ensemble detection is theoretically implemented in linear radiometer calibration to characterize different types of gain processes. In Section V, several simulations are demonstrated as validation studies. Finally, in Section VI, the main conclusions are summarized and future works are discussed.

## II. Ensemble Detection

**Fig. 1.**
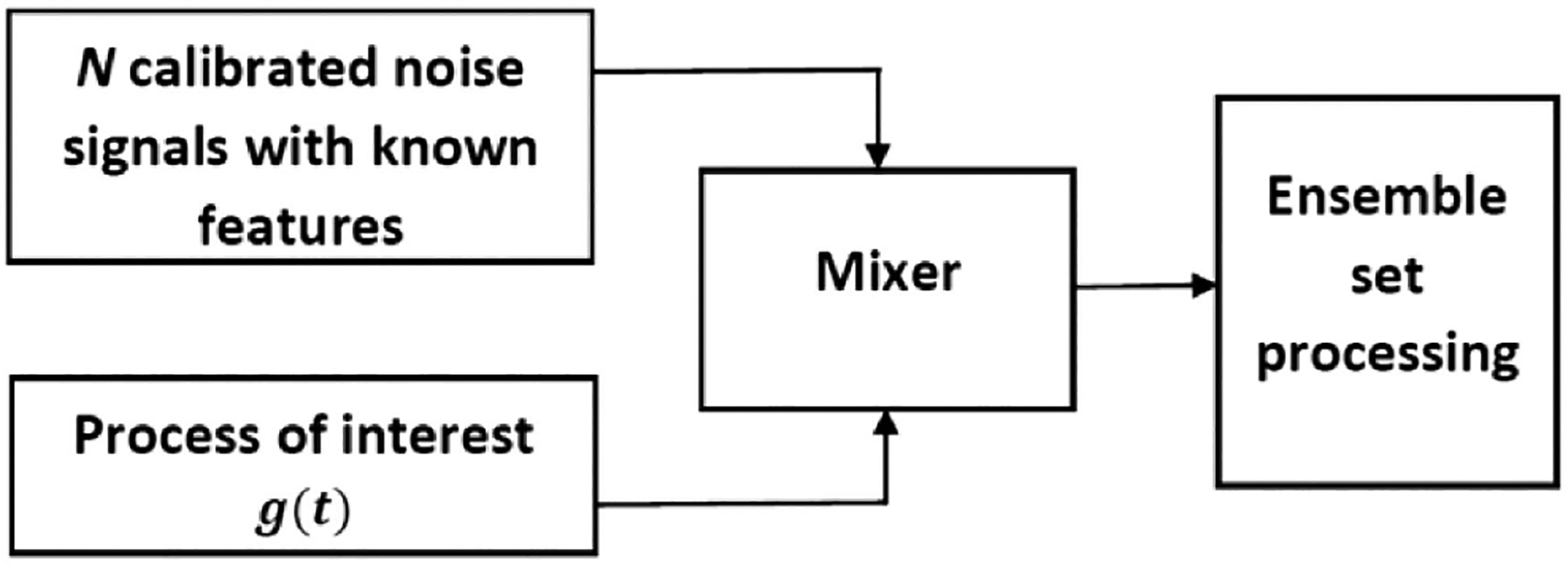
Ensemble detection. An ensemble set for the random process, *g*(*t*), is generated by mixing the process with calibrated noise signals with known features. Then, the ensemble set is processed to retrieve statistical properties of the random process.

The analysis of nonstationary random processes would not be possible by examining their realizations individually. The ensemble detection technique utilizes a noise-assisted methodology in which ensemble sets for random processes are formed by combining their realizations mixed with calibrated noise with known characteristics, as shown in Fig. 1. Then, statistical processing and analyses may be applied on the ensemble set. Thus, ensemble detection may be used to characterize nonstationary random processes for which current statistical approaches do not offer sufficient descriptions, as the ensemble set, unlike a single realization, provides a full picture of the features of the processes in both time and ensemble domains. In Section IV, the theoretical basis for the application of ensemble detection in radiometer calibration is discussed in detail.

## III. Radiometer Calibration

**Fig. 2.**
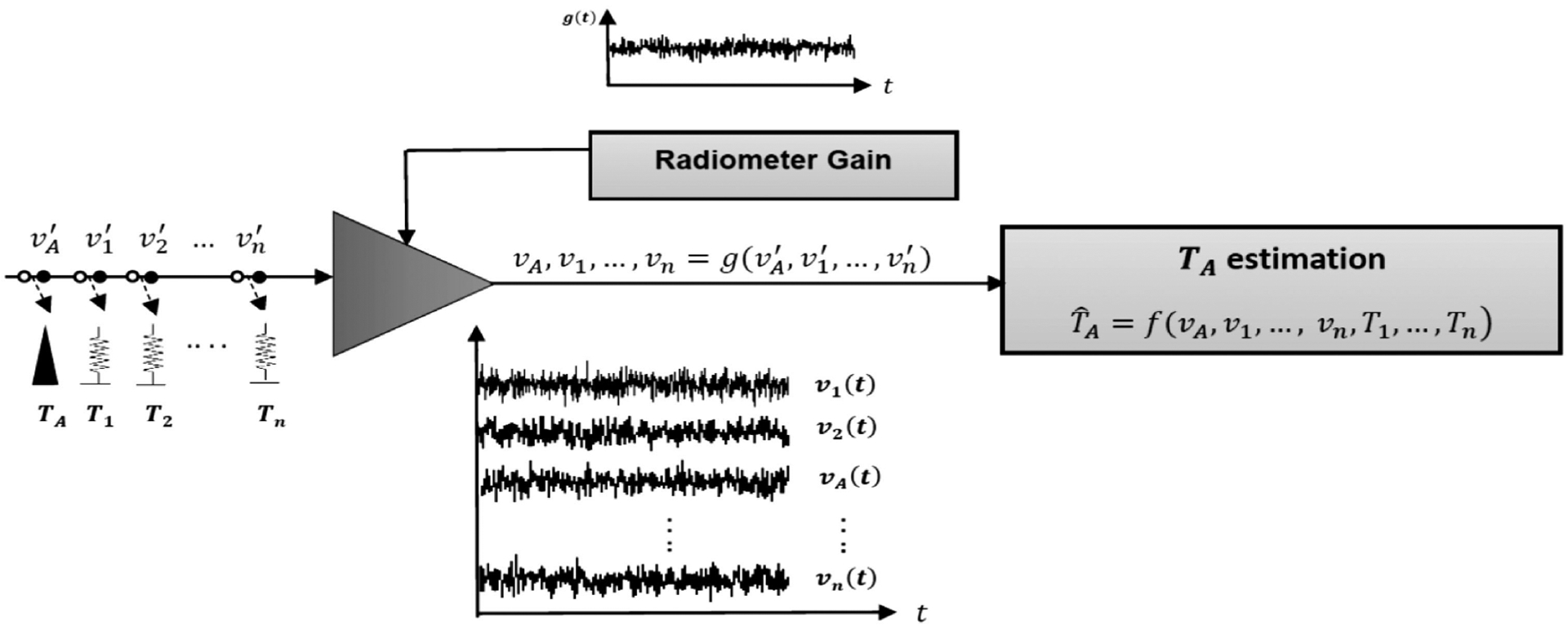
Radiometer calibration scheme with *n* calibration targets. Notice the time series of postgain voltages *v*_*i*_(*t*) form an ensemble set for the random process, which defines the radiometer gain, *g*(*t*).

The received power captured by the radiometer antenna is converted to antenna temperature through the calibration process, as shown in Fig. 2. Radiometric resolution is defined as the minimum change in the input signal level that can be resolved at the calibrated radiometer output. The classic definition of ideal radiometric resolution for a constant gain radiometer is defined in [3], [5], and [26] as follows:

(1) $$\sigma_{T_{A}}≅\frac{T_{{\text{sys}}_{A}}}{\sqrt{B\tau }}$$

where $T_{{\text{sys}}_{A}}$ denotes the system temperature when the antenna temperature is measured, *B* is the predetection bandwidth, and *τ* is the postdetection integration time. When the radiometer gain is not constant, a wide sense stationary process equation (1) becomes [24]

(2) $$\sigma_{T_{A}}≅T_{{\text{sys}}_{A}}{\left(\frac{1}{B\tau }+{\left(\frac{\Delta G}{G}\right)}^{2}\right)}^{1/2}$$

where *G* and Δ*G* represent the mean and standard deviation of the gain power. Now, consider a set of measurements performed by a radiometer system with *n* calibration targets as follows:

(3) $$v_{a}=gT_{A}+b+\varepsilon_{a}\;v_{i}=gT_{i}+b+\varepsilon_{i}\;\forall i\in \{1,\ldots ,n\}$$

where *g* and *b* are the mean gain and offset of the radiometer, *T*_*A*_ is the calibrated antenna temperature, and *T*_*i*_’s are the calibration targets’ temperatures. *v*_*a*_ and *v*_*i*_’s are the output voltages associated with the antenna and calibration targets, respectively, and form an ensemble set for the radiometer gain, as described in [24]. If the radiometer system is assumed to be linear, the antenna temperature can be estimated using this ensemble set and calibration target temperatures via the least square regression. This estimate, $\hat{T_{A}}$ can be expressed as follows:

(4) $${\hat{T}}_{A}=\left(v_{A}-{\langle v_{i}\rangle }_{n}\right)\frac{\sum_{i=1}^{n}\left(v_{i}-{\langle v_{i}\rangle }_{n}\right)T_{i}}{\sum_{i=1}^{n}{\left(v_{i}-{\langle v_{i}\rangle }_{n}\right)}^{2}}+{\langle T_{i}\rangle }_{n}=f\left(x_{1},\ldots x_{k}\right)$$

where 〈*v*_*i*_〉_*n*_ and 〈*T*_*i*_〉_*n*_ are the average of calibration voltages and temperatures, and *x*’s represent all parameters in the formula. Uncertainty in this estimated antenna temperature is defined as follows [23]:

(5) $$\sigma_{{\hat{T}}_{A}}^{2}=E\left\{{\left({\hat{T}}_{A}-{\bar{T}}_{A}\right)}^{2}\right\}$$

where

(6) $${\bar{T}}_{A}=f(\bar{x_{1}},\bar{x_{2}},\ldots ,\bar{x_{k}},),\;\bar{x_{k}}=E\left\{x_{k}\right\}.$$

In order to evaluate the uncertainty in (5), a multivariate Taylor series expansion about the mean value of each random variable is performed on (4). Substituting the series expansion into (5) leads to the law of propagation of uncertainty given by [27]. Assuming the estimator is well approximated by a linear expansion for values of anticipated fluctuations, the series can be truncated at the second term. Thus

(7) $$\sigma_{{\hat{T}}_{A}}^{2}=\sum_{m=1}^{k}\sigma_{x_{m}}{}^{2}f_{x_{m}}{}^{2}+2\sum_{m<n}f_{x_{m}}f_{x_{n}}\text{cov}\left(x_{m}x_{n}\right)$$

where

(8) $$f_{x_{m}}={\frac{\partial }{\partial x_{m}}f\left(x_{1},x_{2},\ldots ,x_{k}\right)|}_{x_{1}=\bar{x_{1}},x_{2}=\bar{x_{2}},\ldots ,x_{k}=\bar{x_{k}}}.$$

Equation (7) is a comprehensive uncertainty definition, which includes both the radiometric resolution and uncertainty due to the calibration process, i.e., uncertainties associated with calibration target measurements [23]. Therefore, using this parameter, it is possible to define a figure of merit for radiometric sensitivity (instead of radiometric resolution described in (1) and (2), which do not include additional uncertainties due to calibration) and facilitate international standards (SI) traceability for radiometry. Furthermore, it provides a standard for the intercalibration of multiple radiometers independent of their calibration structures. Thus, ensemble detection considers (7) as the uncertainty in the antenna temperature estimate, as described in Section IV, rather than focusing on radiometric resolution.

## IV. Theory of Application of Ensemble Detection in Radiometer Calibration

In this section, the application of ensemble detection in radiometer calibration is theoretically explained for three different types of radiometer gain in terms of their temporal stationarity: strictly, weakly, and nonstationary radiometer gain.

### A. Strictly Stationary Radiometer Gain (Gaussian Process)

If it is assumed that the measured pregain voltages, ${v^{\prime }}_{i}$ parameters in Fig. 2, are: 1) stationary Gaussian random processes, ${v^{\prime }}_{i}\sim N\left(T_{{\text{sys}}_{i}},\sigma_{T_{i}}\right)$, where $T_{{\text{sys}}_{i}}$ and $\sigma_{T_{i}}$ are the radiometer system temperature and the ideal resolution associated with the *i*th calibration target measurements, whose samples are independent and identically distributed (IID) Gaussian random variables; and 2) independent of the gain, *g* and among themselves, (7) can be analytically solved for the case of a stationary Gaussian gain *g* ~ *N*(*μ*_*g*_, *σ*_*g*_). In such a case, the variance values in the first term become (assuming no ambiguity present in the knowledge of *T*_*i*_’s)

(9) $$\sigma_{v_{i}}^{2}=\sigma_{T_{i}}{}^{2}\sigma_{g}^{2}+\sigma_{T_{i}}{}^{2}\mu_{g}^{2}+\sigma_{g}^{2}T_{{\text{sys}}_{i}}{}^{2}$$

where

(10) $$v_{i}=g{v^{\prime }}_{i}\;E\left\{v_{i}\right\}=\mu_{g}T_{{\text{sys}}_{i}}$$

and the covariances, cov(*x*_*m*_, *x*_*n*_), in the second term of (7) are

(11) $$\text{cov}\left(v_{j<i}\left(t_{j}\right),v_{i}\left(t_{i}\right)\right)=\sigma_{g}^{2}T_{{\text{sys}}_{j<i}}\left(t_{j}\right)T_{{\text{sys}}_{i}}\left(t_{i}\right)\delta \left(t_{j}-t_{i}\right)$$

where *t*_*j*_ and *t*_*i*_ are the observation times of the calibration targets associated with *v*_*j*_ and *v*_*i*_, and *δ*(.) denotes the Dirac delta function [see Appendix A for the derivation of the expressions in (11)]. Thus, if the uncertainty in antenna temperature is given or measured, the parameters of the gain process can be retrieved by solving (7).

### B. Weakly Stationary Radiometer Gain (AR(1) Process)

Assuming conditions 1) and 2) described in Section IV-A are still satisfied, (7) can also be analytically solved for gain characterization if the radiometer gain is described by weakly stationary AR(1) processes, i.e.,

(12) $$g\;(t)=\beta_{0}+\beta_{1}g\;(t-1)+\epsilon_{g}$$

where *ϵ*_*g*_ ~ *N*(0, *σ*_*ϵ*_), *β*_0_ is a constant, and *β*_1_ is the coefficient, which determines the correlation between the gain samples. Note that in order to maintain the AR(1) model as a weakly stationary process, the absolute value of *β*_1_ should be strictly less than 1; and when *β*_1_ = 0, the model yields a Gaussian radiometer gain described in Section IV-A. One can also note that the mean and variance of the AR(1) process are *μ*_*g*_ = *β*_0_/(1 − *β*_1_) and $\sigma_{g}^{2}=\sigma_{\epsilon }^{2}/\left(1-\beta_{1}^{2}\right)$, respectively. The covariances in the second term of (7) in the case of an AR(1) radiometer gain become

(13) $$\text{cov}\left(v_{j<i}\left(t_{j}\right),v_{i}\left(t_{i}\right)\right)=\beta_{1}^{\left|t_{j}-t_{i}\right|}\sigma_{g}^{2}T_{{\text{sys}}_{j<i}}\left(t_{j}\right)T_{{\text{sys}}_{i}}\left(t_{i}\right)$$

where *t*_*j*_ and *t*_*i*_ are the observation times of the calibration targets associated with *v*_*j*_ and *v*_*i*_, and |.| denotes the absolute value [28]. See the derivation of the expressions in (13) in Appendix B.

### C. Nonstationary Radiometer Gain

For nonstationary radiometer gain, (7) does not yield a trivial analytical relationship between the statistical properties of the radiometer gain and the calibrated antenna temperature. However, one can still model the radiometer gain as a strictly or weakly stationary process in a specific calibration scheme. Given a calibration setup, (7) can be solved as if the radiometer gain was strictly or weakly stationary, and statistical parameters for equivalent gain processes can be retrieved. However, unlike the previous two cases, these parameters would change as the calibration setup, e.g., calibration or antenna observation times, changes.

## V. Simulations and Discussion

A radiometer system with calibration parameters listed in Table I has been simulated to validate the theoretical study discussed in Section IV. The calibration has been performed in 500 s long calibration windows according to (4) by observing calibration targets at fixed times while changing the antenna observation time within the calibration window. Then, the uncertainty in the estimated antenna temperatures has been calculated and averaged over 30 000 Monte-Carlo trials.

**Fig. 3.**
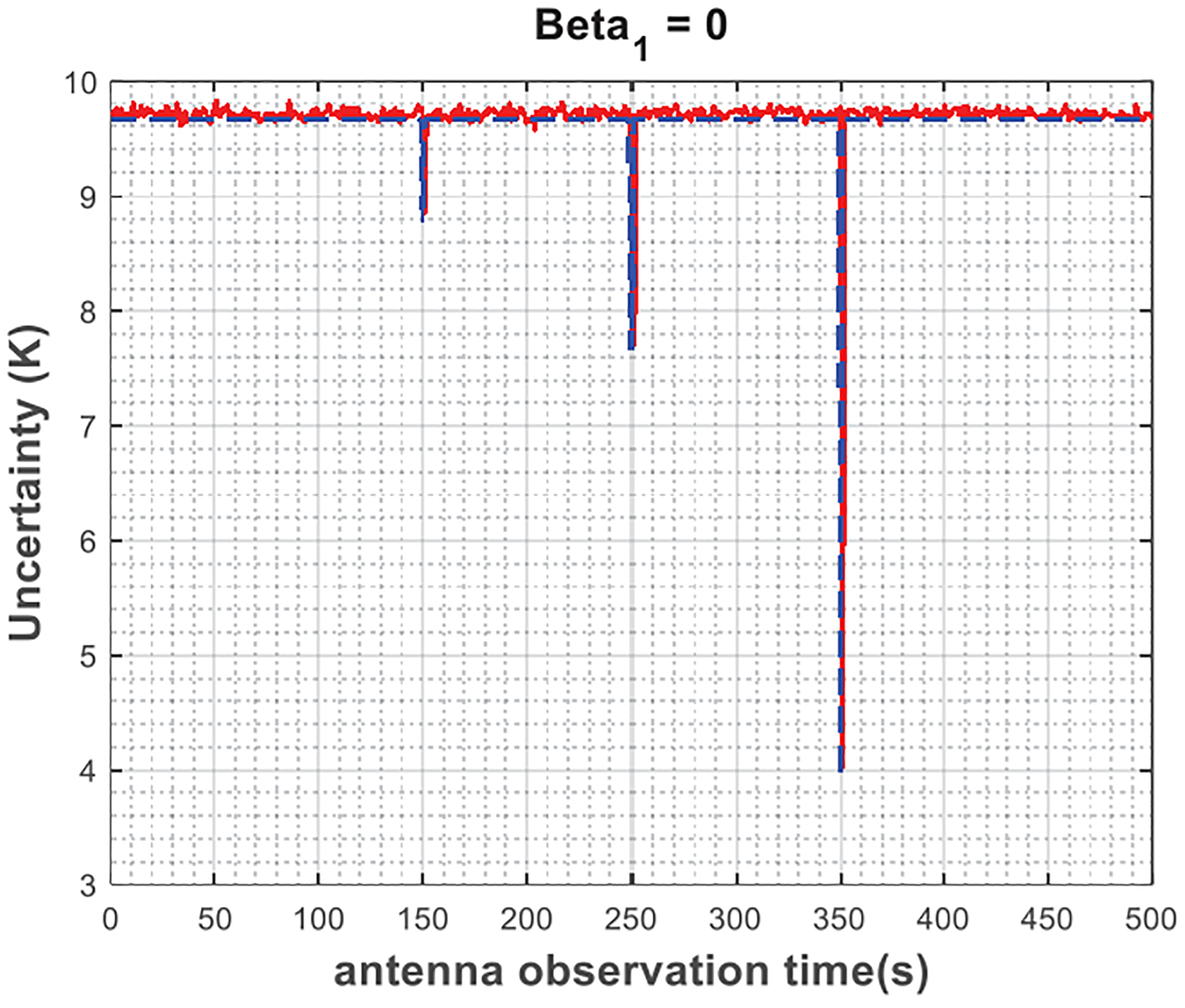
Uncertainty in the estimated antenna temperatures versus antenna observation time for Monte-Carlo simulations (red) and analytical model (blue) when the radiometer gain is strictly stationary Gaussian process.

**Fig. 4.**
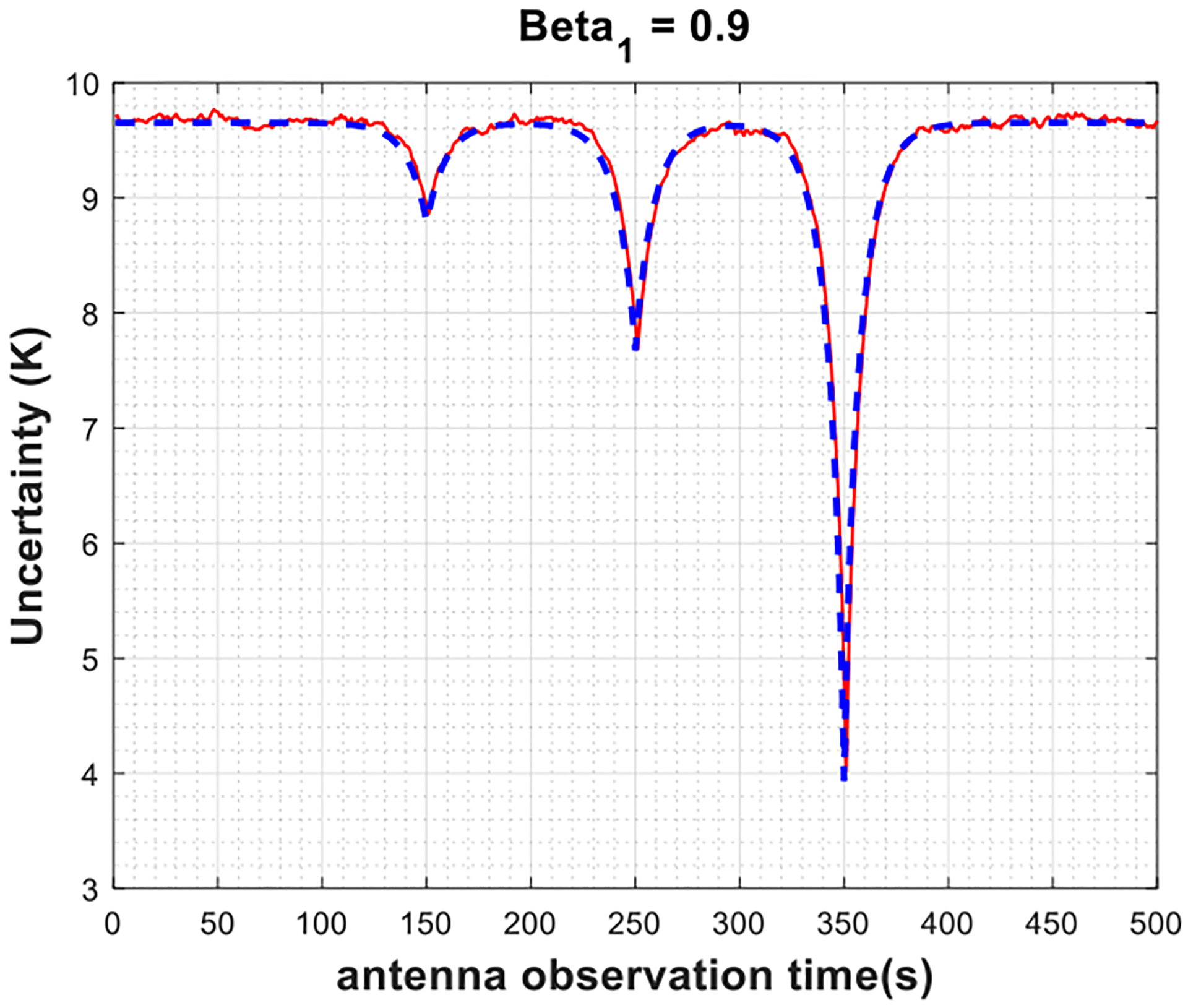
Uncertainty in the estimated antenna temperatures versus antenna observation time for Monte-Carlo simulations (red) and analytical model (blue) when the radiometer gain is weakly stationary AR(1) process with *β*_1_ = 0.9.

**Fig. 5.**
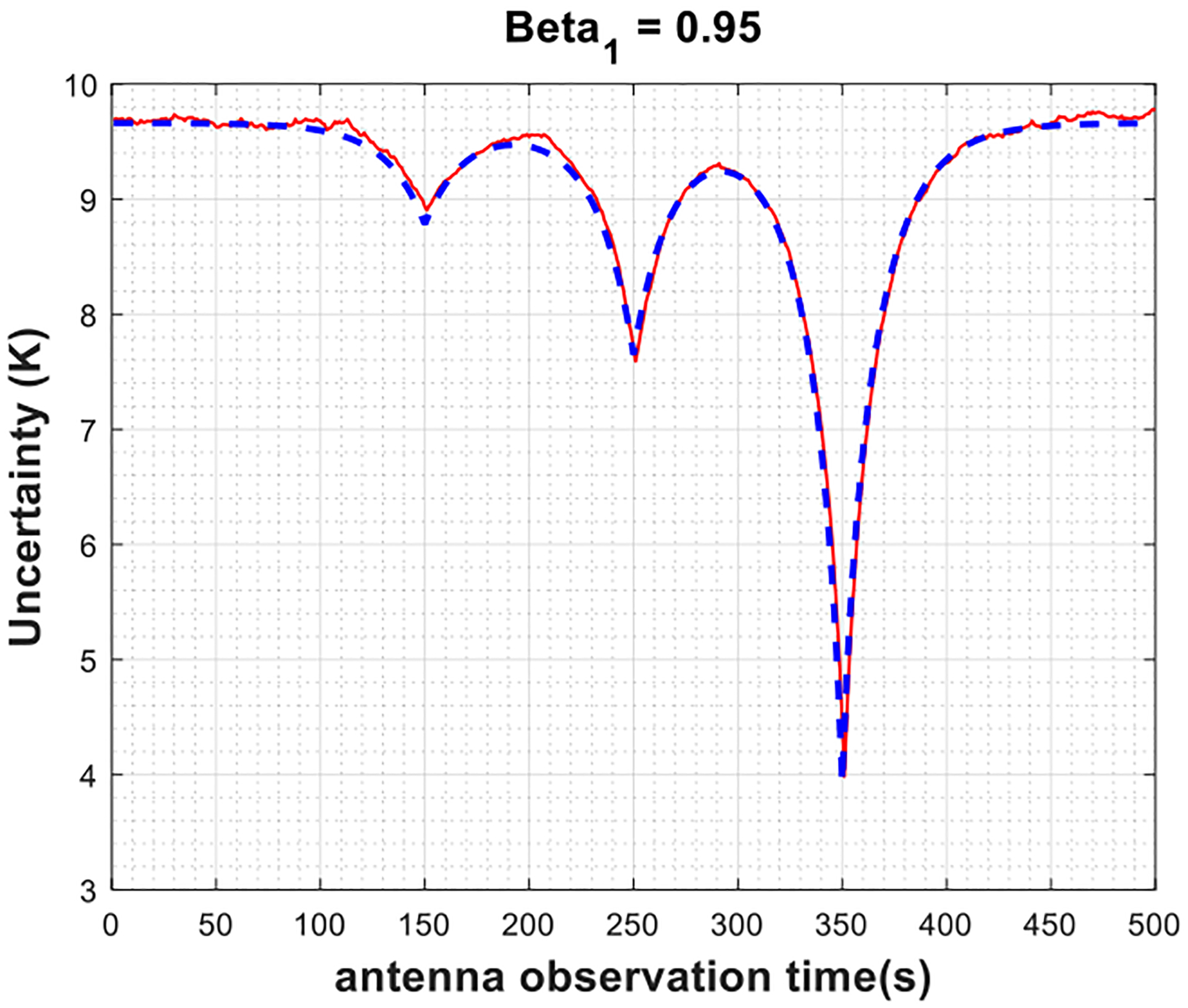
Uncertainty in the estimated antenna temperatures versus antenna observation time for Monte-Carlo simulations (red) and analytical model (blue) when the radiometer gain is weakly stationary AR(1) process with *β*_1_ = 0.95.

Moreover, the analytical uncertainty is also plotted with the same set of input parameters based on (7) by plugging in the corresponding covariance values from (11) and (13) for Gaussian and AR(1) radiometer gain processes, respectively. Figs. 3–5 show the results assuming an AR(1) radiometer gain process for different values of *β*_1_ varying in the interval of [0, 1) (note that *β*_1_ = 0 indicates a Gaussian radiometer gain, and only the steady state of the autoregressive processes is considered). First, the plots illustrate that the Monte-Carlo simulations agree with analytical derivations; thus, validate the theoretical descriptions given in Section IV. As shown in Fig. 3, for strictly stationary Gaussian radiometer gain, the uncertainty in the antenna temperature estimations is independent of the antenna measurement time with respect to the calibration look times. The only exception happens when the antenna is observed at the exact same time with a calibration target, as expected from the Dirac delta functions in the covariance terms in (11), which is indeed impractical for real radiometer systems. In case of AR(1) radiometer gain, as seen in Figs. 4 and 5, the uncertainty changes smoothly as the antenna observation time is brought closer to calibration times such that as *β*_1_ gets larger, the slope of the change decreases. This can be mathematically justified by (13), where the covariance terms exponentially related to the time difference between the antenna and the calibration look times through the term $\beta_{1}^{\left|t_{j}-t_{i}\right|}$. Finally, notice that the average uncertainty levels shown in Figs. 3–5 are higher than the radiometric resolutions defined by (1) and (2), 0.025 K and 8 K, respectively, based on the radiometer parameters provided in Table I. This is due to the fact that calibration measurements introduce the significant amount of uncertainty to the calibrated antenna temperatures, as previously discussed. When antenna and calibration measurements are observed at the same time, or close to one another in time in case of an AR(1) radiometer gain, these uncertainties, thus the uncertainty in the antenna temperature estimates, diminish.

Once the theory is validated using Monte-Carlo simulations, ensemble detection can be used to characterize radiometer gain processes if the basic radiometer parameters, such as those listed in Table I [except the radiometer gain standard deviation assuming that the mean gain is known or can be estimated directly through (3) and (4)], are known and the uncertainty in the estimated antenna temperatures, $\sigma_{\hat{T_{A}}}$, is measured versus antenna observation time, *t*_*a*_, with respect to calibration look times.

### A. Radiometer Gain Characterization

**Fig. 6.**
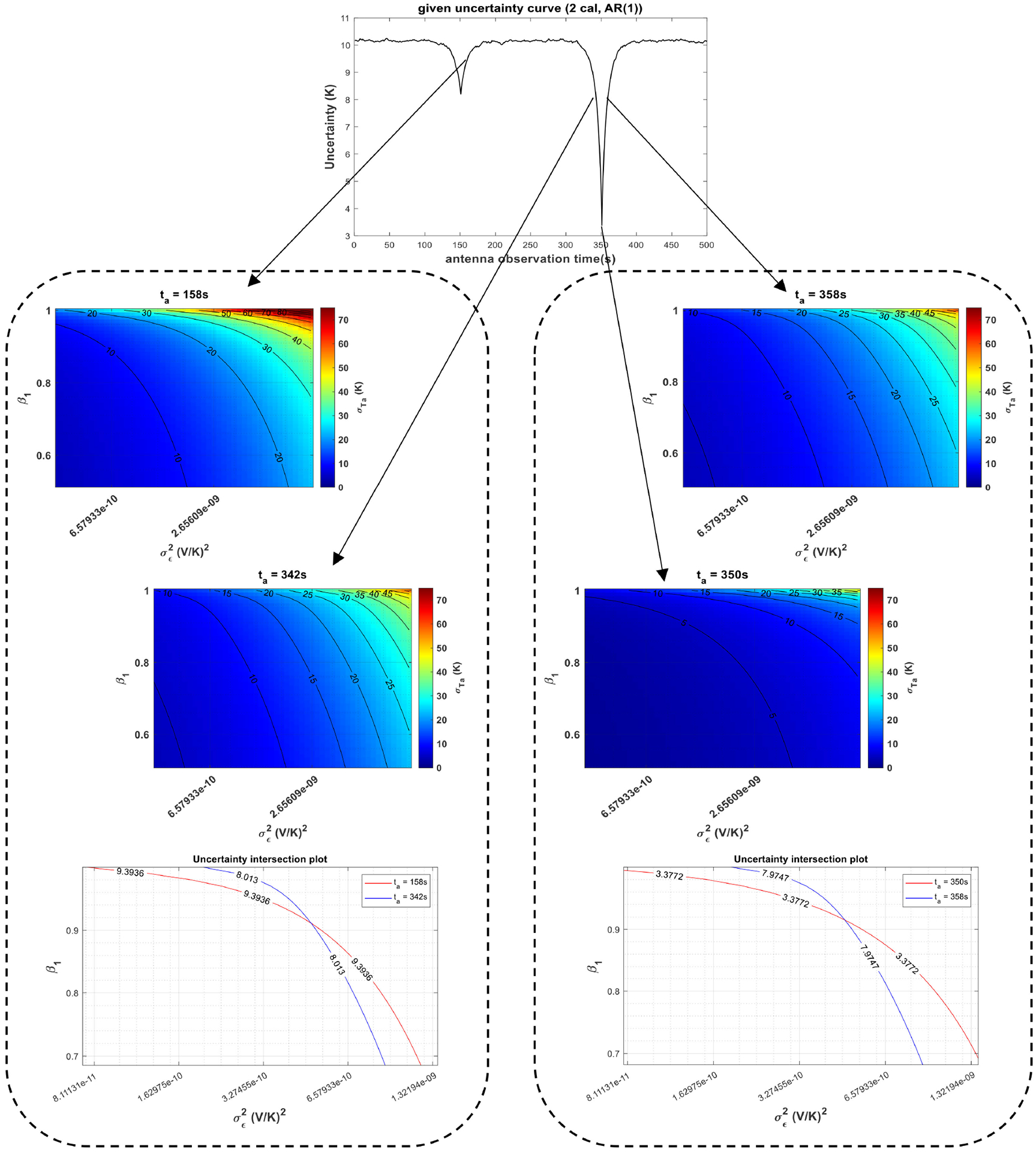
Gain characterization with a given $\sigma_{\hat{T_{A}}}$ versus *t*_*a*_ curve (top) in a two-point calibration structure described in Section V-A when the gain is assumed to follow a weakly stationary AR(1) process. (Middle and bottom) Retrieval of gain parameters (*β*_1_ and $\sigma_{\in }^{2}$) using two different uncertainty pairs at *t*_*a*_ = 158 and 342 s and *t*_*a*_ = 350 and 358 s by solving the set of nonlinear equations via graphing.

**Fig. 7.**
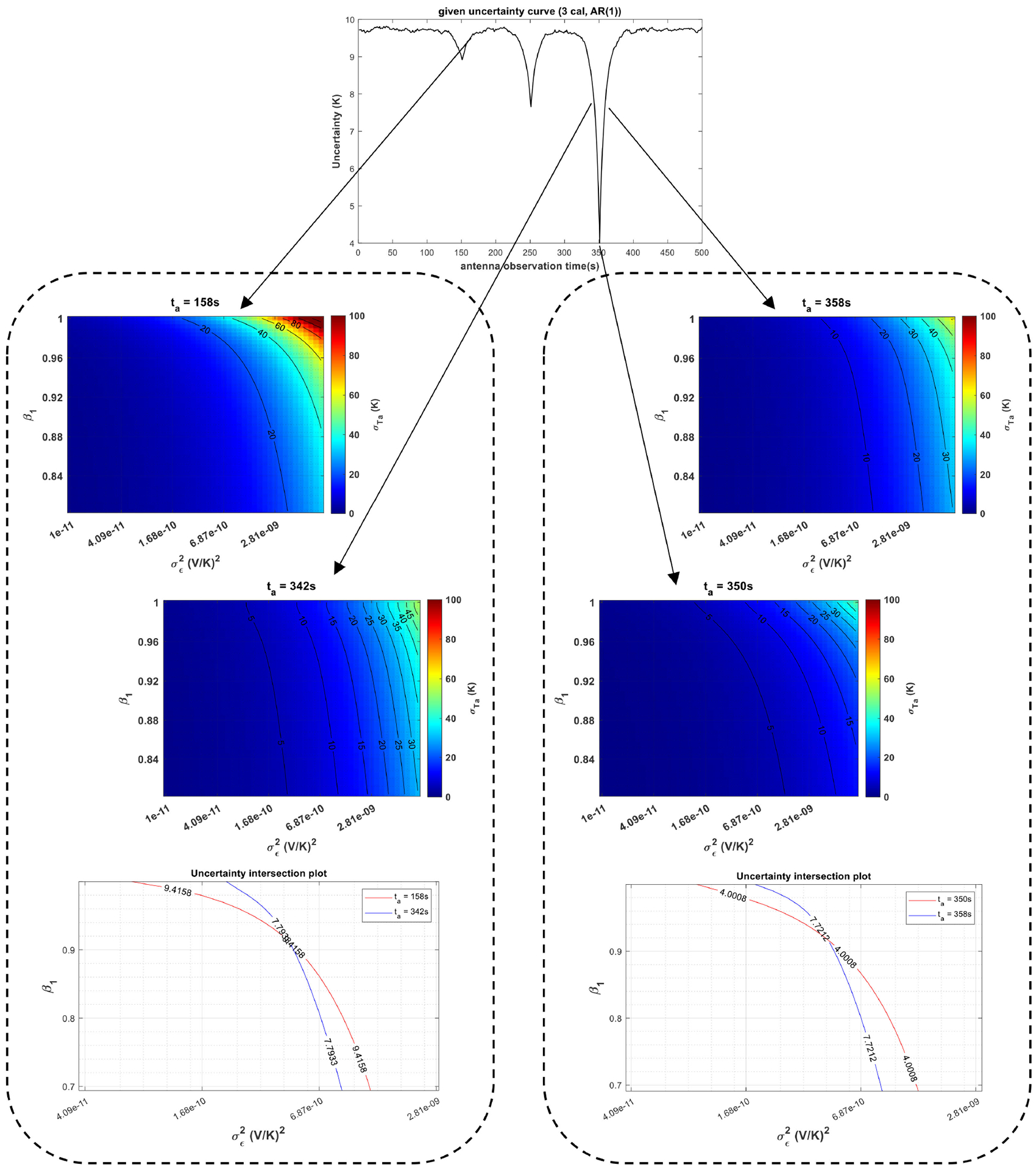
Gain characterization with a given $\sigma_{\hat{T_{A}}}$ versus *t*_*a*_ curve (top) in a three-point calibration structure described in Table I when the gain is assumed to follow a weakly stationary AR(1) process. (Middle and bottom) Retrieval of gain parameters (*β*_1_ and $\sigma_{\in }^{2}$) using two different uncertainty pairs at *t*_*a*_ = 158 and 342 s and *t*_*a*_ = 350 and 358 s by solving the set of nonlinear equations via graphing.

**Fig. 8.**
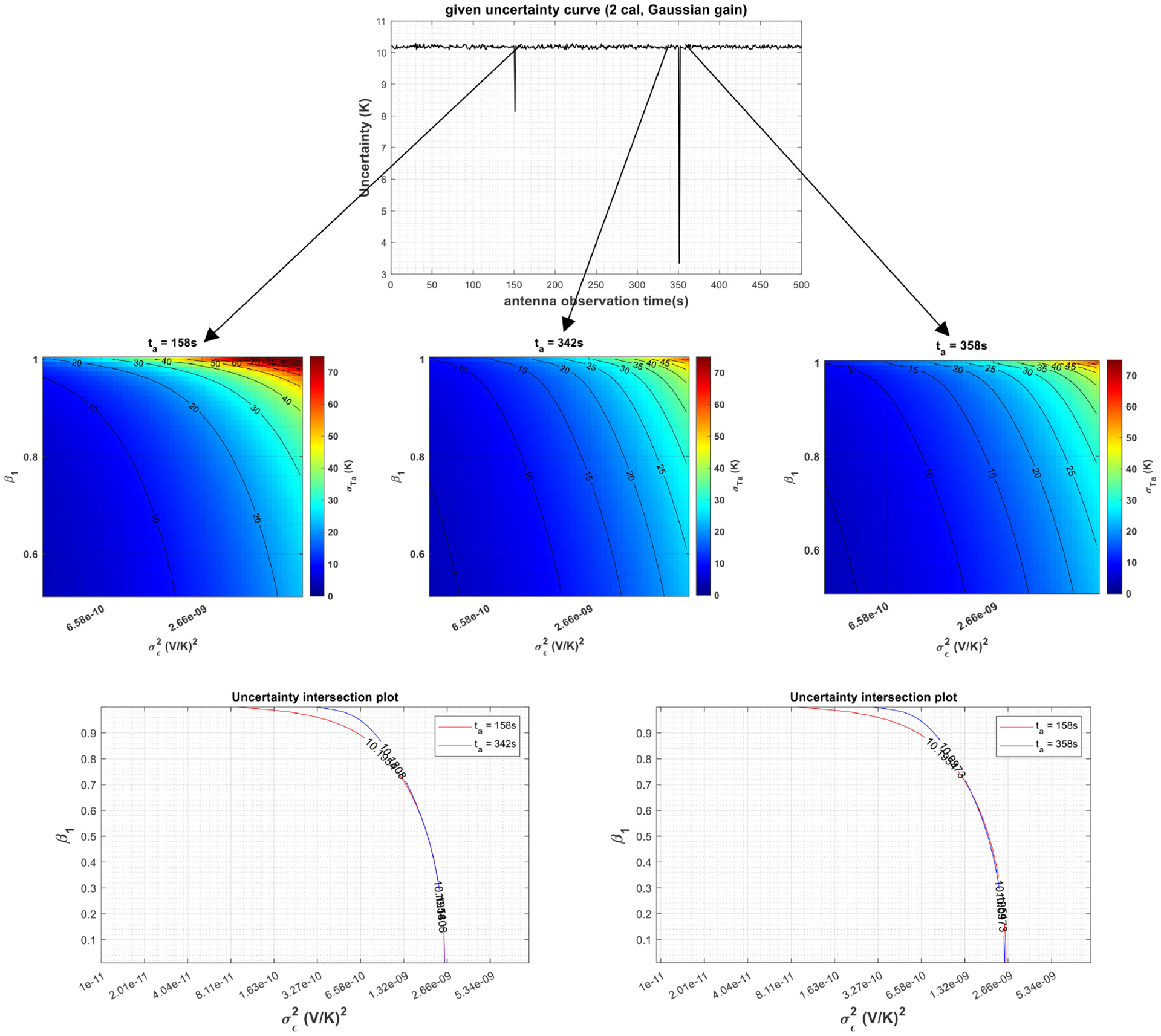
Gain characterization with a given $\sigma_{\hat{T_{A}}}$ versus *t*_*a*_ curve (top) in a two-point calibration structure shown in Fig. 6 when the gain is assumed to follow a strictly stationary Gaussian process. (Middle and bottom) Retrieval of gain parameter ($\sigma_{\in }^{2}$) via graphing by looking at the *β*_1_ = 0 points in the bottom graphs.

Given a specific $\sigma_{\hat{T_{A}}}$ versus *t*_*a*_ curve in a known calibration structure and assuming that the radiometer gain is described by an AR(1) process, as defined in (12), the gain parameters, i.e., the constant value *β*_0_, the correlation coefficient between the gain samples *β*_1_, as well as the variance of white noise $\sigma_{\epsilon }^{2}$ can be retrieved using the theory presented in Section IV. Figs. 6–8 illustrate a methodology for such retrieval. To retrieve *N* unknowns in the gain model, first, at least *N* points with different uncertainty values are selected from the curve. These uncertainty values provide *N* independent nonlinear expressions through (7), which establish the relationship between the gain parameters and the uncertainty in the antenna temperature estimates. Note that these expressions are analytically solvable for an AR(1) process, as explained in Section IV; thus, the solution of this set of *N* equations provides the retrieved gain parameters.

The retrieval depicted in Fig. 6 is for a radiometer system with an AR(1) gain, where *β*_1_ = 0.9. Radiometer parameters are the same as Table I except the fact that only two calibration targets with temperatures 200 K and 350 K are observed at 150 s and 350 s, respectively, in a 500 s calibration window, and the antenna temperature is assumed to be 300 K. It should be noted that since *μ*_*g*_ is known, *β*_0_ and *β*_1_ are not independent, as shown in Section IV-B; so, *β*_1_ and $\sigma_{\epsilon }^{2}$ are the only unknown parameters to retrieve to characterize the radiometer gain. Thus, *N* = 2, the number of uncertainty values should suffice. Two pairs of $\sigma_{\hat{T_{A}}}$ values at *t*_*a*_ = 158 and 342 s and *t*_*a*_ = 350 and 358 s are selected, as shown in Fig. 6. Nonlinear expressions for these pairs are solved for *β*_1_ and $\sigma_{\epsilon }^{2}$ by graphing. The solutions for both pairs yield to the same values for *β*_1_ and $\sigma_{\epsilon }^{2}$, approximately 0.9 and 4.75 × 10^−10^ (V/K)^2^, respectively. Thus, the retrieval is accurate ($\sigma_{\epsilon }^{2}$ and *β*_1_ = 0.9 would produce an AR(1) gain process with standard deviation $\sigma_{g}=\sqrt{\sigma_{\epsilon }^{2}/\left(1-\beta_{1}^{2}\right)}=0.00005\;(\text{V}/\text{K})$, the value listed in Table I) and independent of the uncertainty pairs selected.

Fig. 7 shows the gain characterization procedure for a radiometer system with the same gain process as the previous example and three-point calibration structure, as listed in Table I. Uncertainty pairs are also picked at the same time as the previous example for retrieval and very similar values are obtained as the estimated gain parameters. Thus, the retrieved gain parameters do not change over different selections of *t*_*a*_ pairs as well as different calibration structures, which demonstrates the stationarity of the underlying gain process, at least in a weakly sense.

Finally, Fig. 8 shows the gain characterization when the gain is assumed to follow a Gaussian process and the calibration is performed with two calibration targets. Since the level of uncertainty in the estimated antenna temperatures is expected to be independent of the antenna observation time in such a strictly stationary process, uncertainty selections at *t*_*a*_ = 158 and 342 s, and *t*_*a*_ = 158 and 358 s would yield to similar nonlinear equations. On the other hand, since the value of *β*_1_ is zero in case of a Gaussian radiometer gain, the only unknown parameter would be the $\sigma_{\epsilon }^{2}$, which can be retrieved with only one equation. Consequently, the retrieved $\sigma_{\epsilon }^{2}$ would be the gain variance $\sigma_{g}^{2}$ which should be approximately 2.5 × 10^−9^ (V/K)^2^ for the assumed standard deviation *σ*_*g*_ = 0.00005 (V/K). As shown in Fig. 8, the retrieved noise variance is accurate and almost the same for the two selected antenna measurement time pairs; therefore, it verifies the retrieval approach and the stationarity of radiometer gain.

**Fig. 9.**
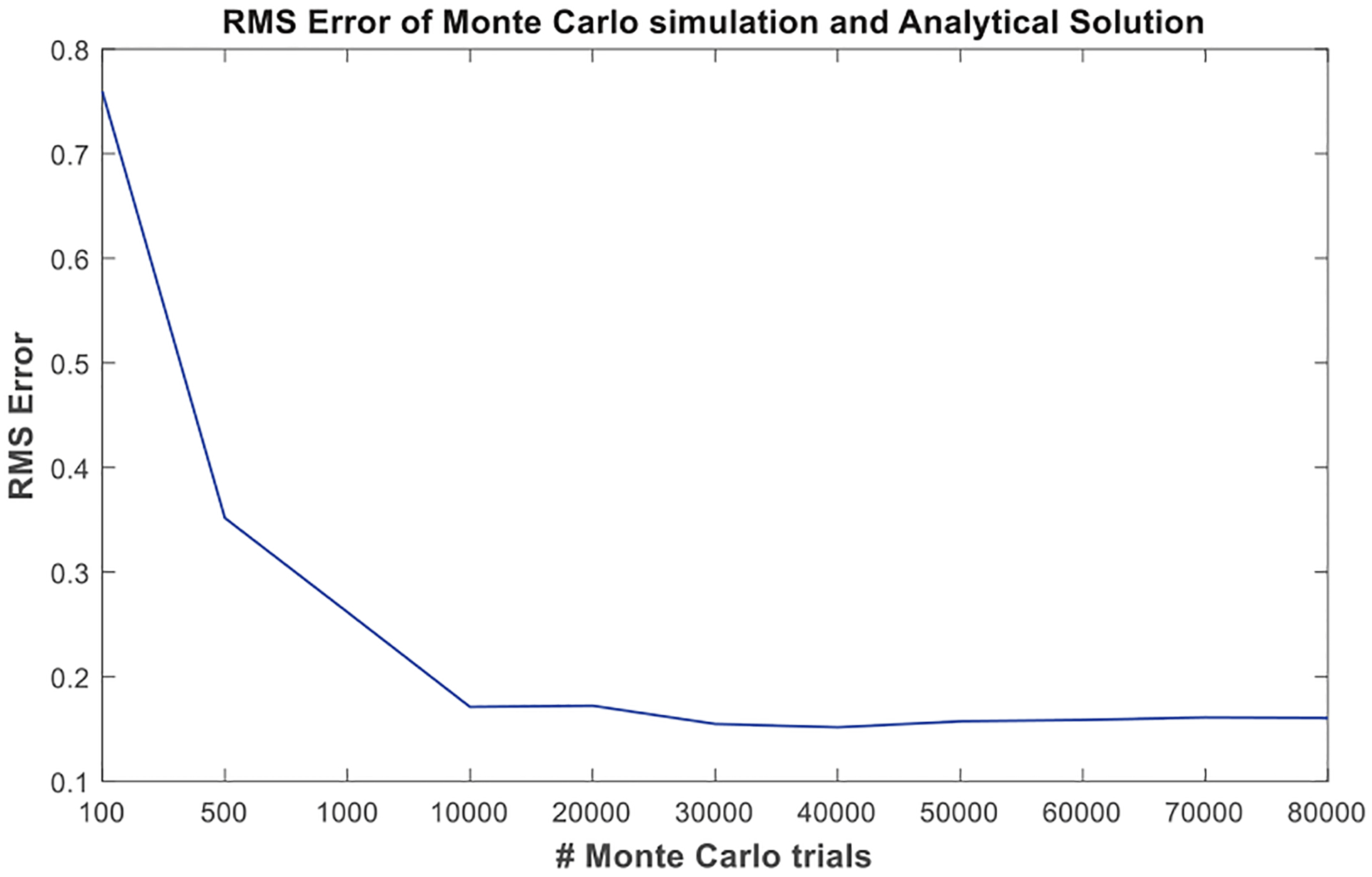
Retrieval error, i.e., rms difference between the retrieved analytical and the given Monte-Carlo $\sigma_{\hat{T_{A}}}$ versus *t*_*a*_ curves versus the number of Monte-Carlo trials.

It is important to note that the accuracy of the gain characterization explained in this article depends on the accuracy of the Monte-Carlo simulations, or in real implementations, the accuracy of the measured uncertainty in antenna temperatures. In Figs. 6–8, for instance, 30 000 trials have been used. As the number of trials in Monte-Carlo simulations or the number of antenna temperature measurements in real experiments increases, the uncertainty curves converge to the analytical calculations described in Section IV; thus, the retrievals become more accurate. Fig. 9 illustrates this by plotting the root-mean-square (rms) error in retrievals versus the number of trials used in Monte-Carlo simulations. The error, here, is defined as the error between the given $\sigma_{\hat{T_{A}}}$ versus *t*_*a*_ curves as the result of Monte-Carlo simulations and the analytically reconstructed $\sigma_{\hat{T_{A}}}$ versus *t*_*a*_ curves using the retrieved gain parameters. As expected, the error decreases as the number of trials increases.

### B. Nonstationary Radiometer Gain

**Fig. 10.**
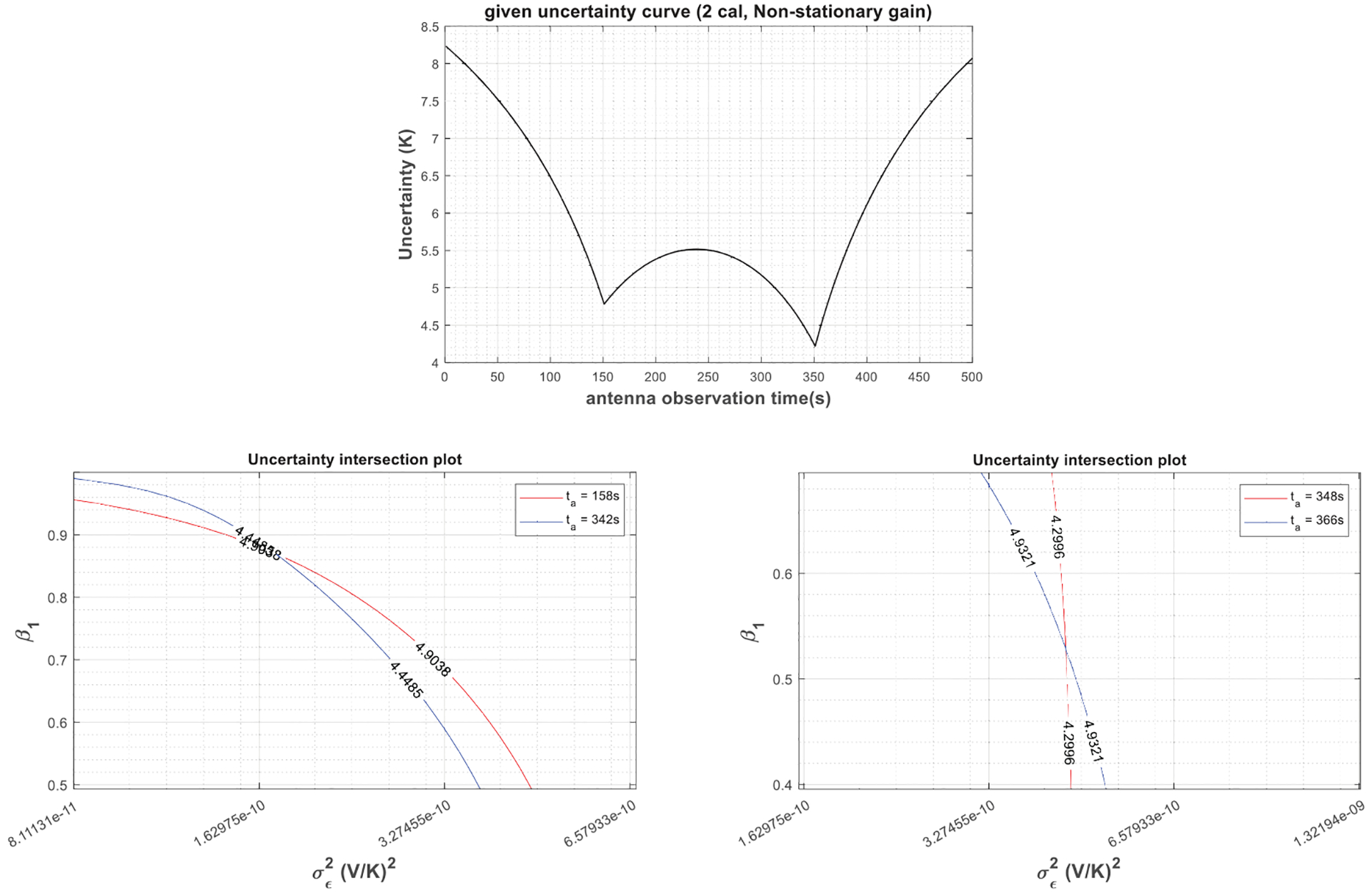
Gain characterization with a given $\sigma_{\hat{T_{A}}}$ versus *t*_*a*_ curve (top) in a two-point calibration structure shown in Fig. 6 when the gain is assumed to follow a nonstationary process. (Middle and bottom) Due to nonstationarity, different gain parameters (*β*_1_ and $\sigma_{\in }^{2}$) have been retrieved when two different uncertainty pairs at *t*_*a*_ = 158 and 342 s and *t*_*a*_ = 348 and 366 s were used to solve the set of nonlinear equations via graphing.

**Fig. 11.**
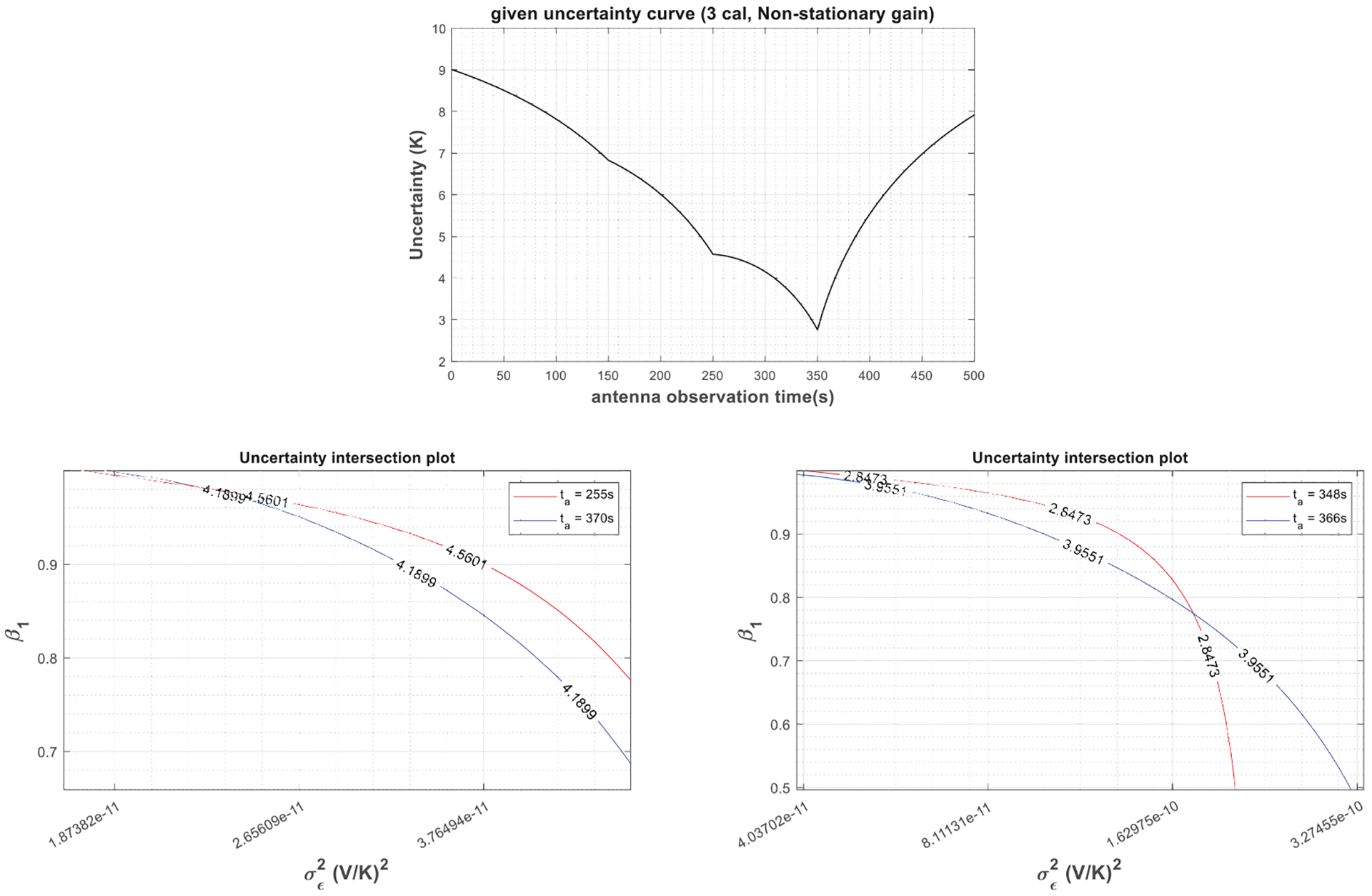
Gain characterization with a given $\sigma_{\hat{T_{A}}}$ versus *t*_*a*_ curve (top) in a three-point calibration structure given in Table I when the gain is assumed to follow a nonstationary process. (Middle and bottom) Due to nonstationarity, different gain parameters (*β*_1_ and $\sigma_{\in }^{2}$) have been retrieved when two different uncertainty pairs at *t*_*a*_ = 255 and 370 s and *t*_*a*_ = 348 and 366 s were used to solve the set of nonlinear equations via graphing. Notice that the retrieved parameters using the *t*_*a*_ = 348 and 366 s pair are also different from the retrieved values shown in Fig. 10 using the same antenna observation times; thus, different calibration structures (two point versus three point) yield to different models as well due to nonstationarity.

Theoretical relationships between the uncertainty in estimated antenna temperatures and the radiometer gain parameters described in Section IV are not valid in case of a nonstationary radiometer gain. In fact, in such a case, obtaining an analytical solution to (7) is quite challenging. On the other hand, in a certain calibration scheme, one can assume that the radiometer gain is strictly or weakly stationary, and retrieve an equivalent gain model, which represents the radiometer behavior only in that particular calibration scheme. Fig. 10 shows the uncertainty in the estimated antenna temperatures versus antenna observation time due to a nonstationary radiometer gain in the radiometer system, as illustrated in Fig. 6. To make the gain process nonstationary, the value of *β*_1_ in the AR(1) model has been changed to 1. Two equivalent gain models are retrieved for this calibration scheme, an AR(1) and a Gaussian process. For the equivalent AR(1) model, the retrievals are performed using two antenna observation time pairs *t*_*a*_ = 158 and 342 s and *t*_*a*_ = 348 and 366 s. As demonstrated in the figure and listed in Table II, unlike the previous cases, the parameters of the equivalent gain model depend on the antenna observation time selections. Fig. 11 and Table III give the same retrieval procedure for the three-point calibration setup given in Table I. The equivalent gain parameters still vary with antenna observation time selections; furthermore, retrieved gain parameters using the same antenna observation time pair (*t*_*a*_ = 348 and 366 s) are different for the two-point and three-point calibration schemes. This is expected as nonstationary processes exhibit different characteristics at different observation times and under different measurement methods. Tables IV and V list the retrieved equivalent Gaussian gain parameter *σ*_*g*_ (or *σ*_*ϵ*_) using the above-mentioned two- and three-point calibration schemes, which also lead to similar conclusions.

### C. Impact of Antenna Temperature

**Fig. 12.**
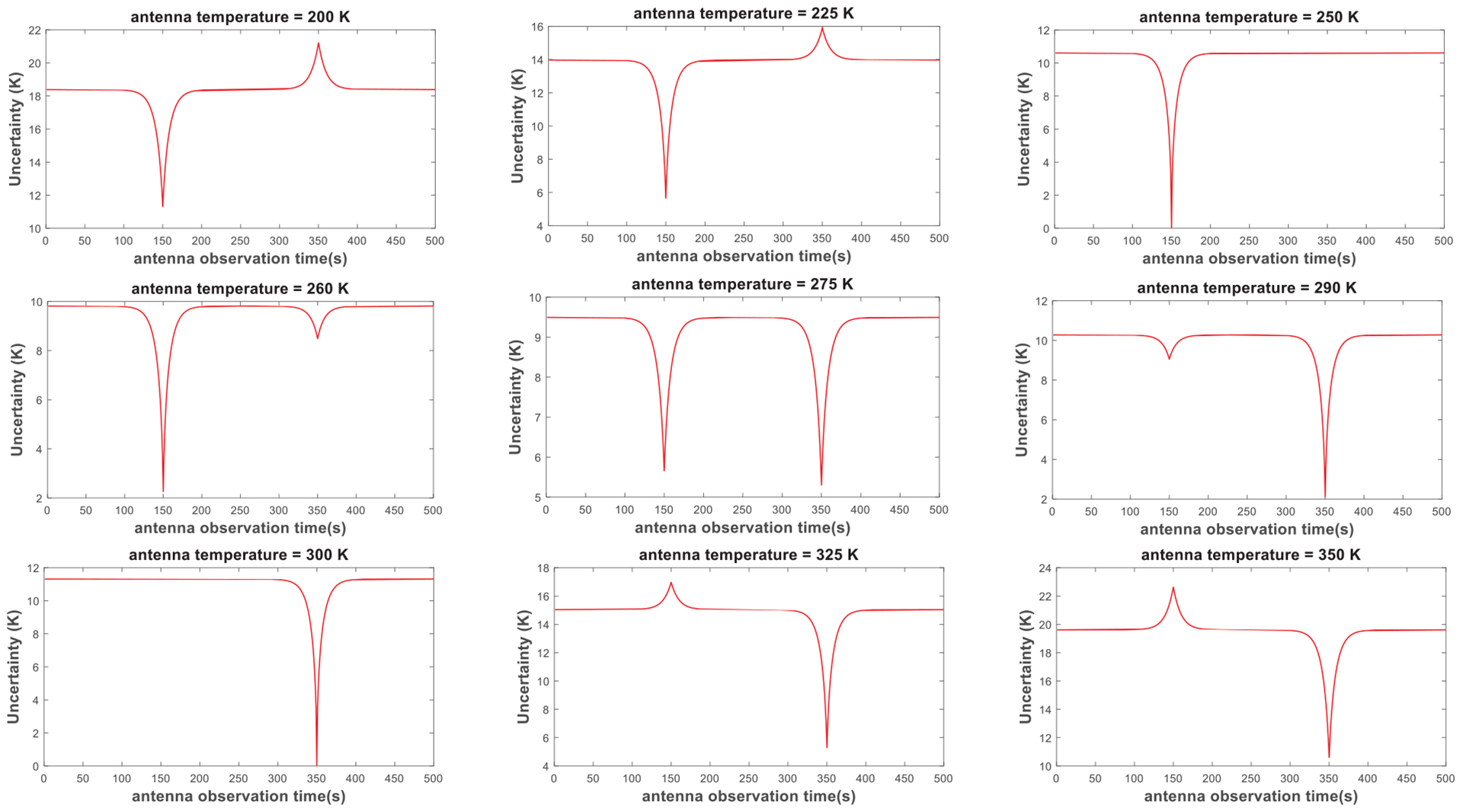
$\sigma_{\hat{T_{A}}}$ versus *t*_*a*_ curves for various antenna temperatures between 200 and 350 K. Calibration temperatures are set as 250 K and 300 K, the rest of the parameters is the same, as demonstrated in Fig. 6. Note the increases in the estimated antenna temperature uncertainties when the antenna temperature is not between the calibration target temperatures.

The studies presented in this article suggest that $\sigma_{\hat{T_{A}}}$ versus *t*_*a*_ curves also depend on the antenna temperature with respect to calibration target temperatures. Fig. 12 shows the analytically calculated antenna temperature uncertainties versus antenna measurement times for the radiometer system depicted in Fig. 6, where the antenna temperature is varied from 200 to 350 K. It can be seen that the amounts of drop (or jump) in uncertainty level near calibration observation times and the overall uncertainty levels are determined by the antenna temperature. When the radiometer gain for the antenna measurements is extrapolated from calibration targets, i.e., the antenna temperature is not between the calibration target temperatures, the overall uncertainty levels increase, and there may be an uncertainty jump when the antenna is observed close in time to the farther calibration target in terms of temperature (see the top left and bottom right curves in Fig. 12). Moreover, if the antenna temperature is similar to one of the calibration target temperatures, the uncertainty is at minimum possible value when the antenna and that target are measured very close to each other in time (see the top right and bottom left curves in Fig. 12). In fact, there is no uncertainty due to the calibration procedure in this case, and the residual uncertainty is only due to the radiometric resolution described in (1). Fig. 12 also demonstrates that a typical radiometer calibration, wherein the antenna temperature is in between the calibration temperatures, provides nearly optimum uncertainty levels irrespective of the antenna measurement time (the middle row in Fig. 12).

## VI. Conclusions and Future Work

In this article, ensemble detection is introduced and applied in a set of radiometer calibration simulations to retrieve the radiometer gain characteristics. In the case of strictly or weakly stationary radiometer gain for which the ensemble set provides an analytical relationship between the statistical properties of the antenna temperature and the radiometer gain, a characterization method is demonstrated to accurately retrieve gain parameters using the uncertainty in antenna temperature estimates versus antenna observation time. The retrieval error diminishes with the number of trials in simulations or the number of measurements in real radiometer experiments. On the other hand, if the radiometer gain is described by a nonstationary process, the same ensemble set can be used to model the radiometer gain as either Gaussian or first-order AR(1) process, where the parameters of these equivalent models depend on the antenna measurement times and the calibration scheme.

As future work, real radiometer measurements will be used in order to further validate the use of ensemble detection to characterize radiometer gain processes. Ensemble detection is applicable to any radiometer system which implements adaptive calibration timing, or stores calibration target and antenna temperatures measurements with relevant timing information for postprocessing calibration. Radiometers with adaptive calibration timings can change time difference between the antenna and the calibration measurements during onboard calibration based on the calibration target availability or to observe critical phenomena in actual time, thus generate $\sigma_{\hat{T_{A}}}$ versus *t*_*a*_ curves to be used in ensemble detection, as explained in Section V. Such radiometers are not very common today but may be developed in the future as developing technologies allow for more intelligent and cognitive systems. Radiometers which store calibration and antenna measurements for postprocessing calibration, on the other hand, are already available for ensemble detection demonstration. Such radiometer systems provide time series of measurements, where antenna observations at different times with respect to certain calibration measurements can be picked to create $\sigma_{\hat{T_{A}}}$ versus *t*_*a*_ curves. Indeed, some studies have already reported experimental $\sigma_{\hat{T_{A}}}$ versus *t*_*a*_ curves generated through real radiometer calibration experiments, where short measurement cycles were treated as different trials over which the uncertainty in antenna temperature estimates at various times were calculated using calibration measurements at fixed times within each cycle (see [29, Fig. 5] and [30, Fig. 17]). Such investigations will be utilized and more analyses will be done with the calibration data measured by real systems to characterize their gain. It is also recognized that gain characterization by selecting specific points from the uncertainty curves, as described in Section V, may lead to multiple gain parameter retrievals as more uncertainty curves can share those points. Thus, to ensure a unique retrieval in future studies, it is planned that the gain characterization will be done by using uncertainty curves in their entirety rather than selecting a number of points from them. Furthermore, in addition to Gaussian and AR(1) processes, additional types of random processes will be incorporated into the ensemble detection studies.

Finally, it should be noted that in this article radiometer systems are assumed to be linear. Impacts of the nonlinear effects on the gain characterization and implementation of the presented methods in nonlinear radiometer systems will be studied in the future. Moreover, it is accepted that the voltage distributions associated with different radiometer calibration targets are separate from one another enough that (4) does not have poles.

## Figures and Tables

**TABLE I:** Simulated Radiometer Parameters

Parameter	Value
Antenna temperature (K)	300
Number of calibration targets	3
Calibration temperatures (K)	[200 250 350]
Receiver temperature (K.)	500
Bandwidth (GHz)	1
Integration time (s)	1
Calibration window length (s)	500
Calibration observation times (s)	[150 250 350]
Mean radiometer gain (V/K)	0.005
Radiometer gain standard deviation (V/K)	0.00005

**TABLE II:** Retrieved AR(1) Model Parameters for a Nonstationary Radiometer Gain Process in a Two-Point Calibration Scheme

t_a_ pair (s)	158, 342	348, 366
***β***_***1***_	0.87	0.52
**$\sigma_{\in }^{2}\;{\left(V/K\right)}^{2}$**	~1.75×10^−10^	~4.33× 10^−10^

**TABLE III:** Retrieved AR(1) Model Parameters for a Nonstationary Radiometer Gain Process in a Three-Point Calibration Scheme

t_a_ pair (s)	255, 370	348, 366
***β***_***1***_	0.98	0.77
**$\sigma_{\in }^{2}\;{\left(V/K\right)}^{2}$**	~2.31× 10^−11^	~1.75× 10^−10^

**TABLE IV:** Retrieved Gaussian Model Parameters for a Nonstationary Radiometer Gain Process in a Two-Point Calibration Scheme

t_a_ (S)	100	250	358
**$\sigma_{T_{A}}\;(K)$**	6.4833	5.5052	4.5726
***σ***_***g***_**(= *σ***_***∊***_**) (V/K)**	3.28× 10^−5^	2.75× 10^−5^	2.23× 10^−5^

**TABLE V:** Retrieved Gaussian Model Parameters for a Nonstationary Radiometer Gain Process in a Three-Point Calibration Scheme

t_a_ (S)	100	250	358
**$\sigma_{T_{A}}\;(K)$**	6.7291	3.0928	3.4209
***σ***_***g***_**(= *σ***_***∊***_**) (V/K)**	4.1× 10^−5^	2.95× 10^−5^	1.12× 10^−5^

## Appendix A

### Proof of Equation (11)

In this appendix, the covariance terms in (7), i.e., cov(*x*_*m*_, *x*_*n*_), where *x*’s represent the voltages and temperatures in the calibration equation (4), are derived for Gaussian radiometer gain processes in the following cases.

*Case 1:* When *x*_*m*_ and *x*_*n*_ are observed at different times.

Since all time-series pregain voltages, temperatures, and the radiometer gain whose samples are IID Gaussian random variables are independent of one another

(14) $$\text{cov}\;\left(v_{i}\;\left(t_{i}\right),\;T_{i}\left(t_{i}\neq t_{i}\right)\right)=0$$

(15) $$\text{cov}\;\left(v_{j<i}\left(t_{j}\right),\;v_{i}\left(t_{i}\neq t_{j}\right)\right)=0$$

(16) $$\text{cov}\;\left(T_{j<i}\;\left(t_{j}\right),\;T_{i}\left(t_{i}\neq t_{j}\right)\right)=0.$$

*Case 2:* When *x*_*m*_ and *x*_*n*_ are observed at the same time

(17) $$\text{cov}\;\left(T_{j<i}\left(t_{j}\right),\;T_{i}\left(t_{i}=t_{j}\right)\right)=0$$

as different calibration target temperatures are independent processes, and

(18) $$\text{cov}\;\left(v_{j<i}\left(t_{j}\right),\;v_{i}\left(t_{i}=t_{j}\right)\right)=\sigma_{g}{}^{2}E\left\{T_{{\text{sys}}_{j<i}}\left(t_{j}\right)\right\}\;E\;\left\{T_{{\text{sys}}_{i}}\left(t_{i}=t_{j}\right)\right\}$$

(19) $$\text{cov}\;\left(v_{j}\left(t_{j}\right),\;T_{i}\;\left(t_{i}=t_{j}\right)\right)=0$$

as *v*(*t*) = *g*(*t*)*v*′(*t*) according to (10) in the article and it is assumed that no ambiguity present in the knowledge of *T*_*i*_’s.

From Cases 1 and 2, one can conclude the generic expression for the covariance terms as follows:

(20) $$\text{cov}\;\left(v_{j<i}\left(t_{j}\right),\;v_{i}\left(t_{i}\right)\right)=\sigma_{g}^{2}T_{{\text{sys}}_{j<i}}\left(t_{j}\right)T_{{\text{sys}}_{i}}\left(t_{i}\right)\delta \left(t_{j}-t_{i}\right)$$

where *δ*(.) denotes the Dirac delta function.

## Appendix B

### Proof of Equation (13)

In this appendix, the covariance terms in (7), i.e., cov(*x*_*m*_, *x*_*n*_), where *x*’s represent the voltages and temperatures in the calibration equation (4), are derived for AR(1) gain processes in the following cases.

*Case 1:* When *x*_*m*_ and *x*_*n*_ are observed at different times

(21) $$\text{cov}\;\left(T_{j<i}\left(t_{j}\right),T_{i}\left(t_{i}\neq t_{j}\right)\right)=0$$

(22) $$\text{cov}\;\left(v_{j}\left(t_{j}\right),\;T_{i}\left(t_{i}\neq t_{j}\right)\right)=0$$

as calibration target temperatures are independent processes, whose samples are IID Gaussian random variables, and the gain process is also independent of them, and

(23) $$\text{cov}\;\left(v_{j<i}\left(t_{j}\right),\;v_{i}\left(t_{i}\neq t_{j}\right)\right)=\text{cov}\left(g\left(t_{j}\right),\;g\left(t_{i}\neq t_{j}\right)\right)\;T_{{\text{sys}}_{j<i}}\left(t_{j}\right)T_{{\text{sys}}_{i}}\left(t_{i}\neq t_{j}\right)$$

as *v*(*t*) = *g*(*t*)*v*′(*t*) according to (10) in the article.

*Case 2:* When *x*_*m*_ and *x*_*n*_ are observed at the same time

(24) $$\text{cov}\;\left(T_{j<i}\left(t_{j}\right),\;T_{i}\left(t_{i}=t_{j}\right)\right)=0$$

as different calibration target temperatures are independent processes, and

(25) $$\text{cov}\;\left(v_{j}\left(t_{j}\right),\;T_{i}\left(t_{i}=t_{j}\right)\right)=0\text{cov}(v_{j<i}\left(t_{j}\right),\;$$

(26) $$v_{i}\left(t_{i}=t_{j}\right))=\text{cov}\left(g\left(t_{j}\right),\;g\left(t_{i}=t_{j}\right)\right)\;T_{{\text{sys}}_{j<i}}\left(t_{j}\right)T_{{\text{sys}}_{i}}\left(t_{i}=t_{j}\right)$$

as *v*(*t*) = *g*(*t*)*v*′(*t*) according to (10) in the article and it is assumed that no ambiguity present in the knowledge of *T*_*i*_’s. To calculate cov(*g*(*t*_*i*_), *g*(*t*_*j*_)) in (23) and (26), *g*(*t*) in (12) can be written iteratively as follows:

(27) $$g\;(t)=\beta_{0}\sum_{n=0}^{k-1}\beta_{1}^{n}+\beta_{1}^{k}g\;(t-k)+\sum_{n=0}^{k-1}\beta_{1}^{n}\epsilon_{g}\;(t-n).$$

And this leads to

(28) $$\text{cov}\;(g\;(t),\;g\;(t-k))=\beta_{1}^{k}\sigma_{g}^{2}.$$

As a result, using (23), (26), and (28), the covariance terms in (7) can be written as follows:

(29) $$\text{cov}\;\left(v_{j<i}\left(t_{i}\right),\;v_{j}\left(t_{j}\right)\right)=\beta_{1}^{\left|t_{j}-t_{i}\right|}\sigma_{g}^{2}\;T_{{\text{sys}}_{j<i}}\left(t_{j}\right)\;T_{{\text{sys}}_{i}}\left(t_{i}\right)$$

and (13) is proved.
